# The hallmarks of dietary intervention-resilient gut microbiome

**DOI:** 10.1038/s41522-022-00342-8

**Published:** 2022-10-08

**Authors:** Natalia S. Klimenko, Vera E. Odintsova, Anastasia Revel-Muroz, Alexander V. Tyakht

**Affiliations:** 1 Center for Precision Genome Editing and Genetic Technologies for Biomedicine, Institute of Gene Biology, Russian Academy of Sciences, Moscow, Russia; 2 Atlas Biomed Group - Knomx LLC, Tintagel House, 92 Albert Embankment, Lambeth, London SE1 7TY UK

**Keywords:** Microbial ecology, Microbiome

## Abstract

Maintaining equilibrium of the gut microbiome is crucial for human health. Diet represents an important and generally accessible natural channel of controlling the nutrients supply to the intestinal microorganisms. Although many studies showed that dietary interventions can specifically modulate gut microbiome composition, further progress of the approach is complicated by interindividual variability of the microbial community response. The reported causes of this variability include the baseline microbiome composition features, but it is unclear whether any of them are intervention-specific. Here, we applied a unified computational framework to investigate the variability of microbiome response measured as beta diversity in eight various dietary interventions using previously published 16S rRNA sequencing datasets. We revealed a number of baseline microbiome features which determine the microbiome response in an intervention-independent manner. One of the most stable associations, reproducible for different interventions and enterotypes, was a negative dependence of the response on the average number of genes per microorganism in the community—an indicator of the community functional redundancy. Meanwhile, many revealed microbiome response determinants were enterotype-specific. In Bact1 and Rum enterotypes, the response was negatively correlated with the baseline abundance of their main drivers. Additionally, we proposed a method for preliminary assessment of the microbiome response. Our study delineats the universal features determining microbiome response to diverse interventions. The proposed approach is promising for understanding the mechanisms of gut microbiome stability and improving the efficacy of personalised microbiome-tailored interventions.

## Introduction

Currently, there is lots of evidence for multifaceted interactions between human health and gut microbial community structure. Changes of microbiome composition were observed for diverse diseases including inflammatory bowel diseases^1–4^, colorectal cancer^5,6^, type 2 diabetes mellitus^7,8^ and obesity^9,10^. In addition to the pathologies directly related to the gastrointestinal tract functioning and digestion, the associations with microbiome were also observed for cardiovascular diseases^11,12^ and allergies^13^, as well as disorders affecting the nervous system - such as Parkinson's disease^14^, autism^15^ and Viliuisk encephalomyelitis^16^. The number of associations between disease states and gut microbiome is steadily increasing. Moreover, multiple studies on model animals colonised with defined microbial consortia demonstrated the causality of microbiome changes in regards to the host health disruption^17^.

Two important ecological properties of gut microbiome are its abilities: (1) to maintain its taxonomic composition stability over time and, on the other hand, (2) to alter its composition rapidly under the influence of certain factors, in particular, diet changes. Several studies with a large number of sampled time points per individual showed that, in general, the interindividual variability of the microbiome composition exceeds the intraindividual variability over time^18–20^. On the contrary, short-term changes in diet, administration of medicines or probiotics, as well as onset of an intestinal infection, can cause significant changes in the composition of the colon microbiota in a fairly short time (measured in days)^18^. For at least some of these changes, their directions were consistent across subjects and reproducible in different cohorts. However, there are differences between the subjects in the magnitude of these changes - in other words, the interindividual variability of the microbiome response to intervention^21–23^. The variability was observed for different types of interventions, such as probiotic and prebiotic intake, specific foods, overall dietary pattern changes^24–26^ and faecal microbiota transplantation^27^.

One of the most intriguing challenges in such studies is to identify the possible causes of the observed variability. This issue is interesting from both fundamental and practical perspectives. It can elucidate the ecological mechanisms underlying diversity of gut communities^28^; on the other hand, it can help tailor the optimal intervention type for a particular person targeting health improvements^23,29,30^. In several studies addressing this question, the researchers have suggested that one of the main factors influencing the rate of microbiome response to dietary intervention is the individual prior long-term dietary pattern^26,31^ (Supplementary Table 1). However, in some other studies, no associations with long-term diet were observed^32^. Other factors suggested to affect the magnitude of microbiome response included host gene expression pattern^25^, various metabolic parameters^33^ as well as baseline (initial) microbiome composition (Supplementary Table 1)^24–26,32–45^. Baseline features of gut microbiome composition are outstanding among such factors, as due to the potential of modulating gut community, there is an opportunity to control the individual response in the perspective. The microbiome features shown to influence the degree of response include alpha diversity, relative abundance of specific taxa and metabolic pathways. Some studies showed good performance of the subjects classification into responders and non-responders based on baseline features including microbiome content^32,44^. Moreover, some of the predictions were confirmed mechanistically in interventional experiments using animal models: transplantation of the responders’ microbiome to the animal caused a more pronounced response to the subsequent intervention^46^.

The majority of studies to investigate dependence of gut community response to dietary interventions on its initial state have small sample size (<50 subjects per intervention (Supplementary Table 1)). Small sample size along with the differences in study design and analytical approaches hamper generalisation of the conclusions across these studies. Particularly, it remains unclear whether among the baseline features associated with the microbiome response there are universal for various interventions. In the present study, we have analysed the microbiome response variation and its dependence on the baseline microbiome composition under eight distinct dietary interventions using previously published data on adult subjects. These interventions utilised different ways of microbiome modulation including change of dietary pattern, prebiotics and probiotics intake, as well as intake of products that are believed not to affect microbiome (placebo groups). By applying unified data analysis methods to these studies, we explored the questions of how the microbiome response can be measured, if there are microbiome features associated with the response universal among interventions and whether it is possible to partly predict the response prior to the intervention.

## Results

### The metrics of microbiome response to dietary interventions

**Fig. 1:**
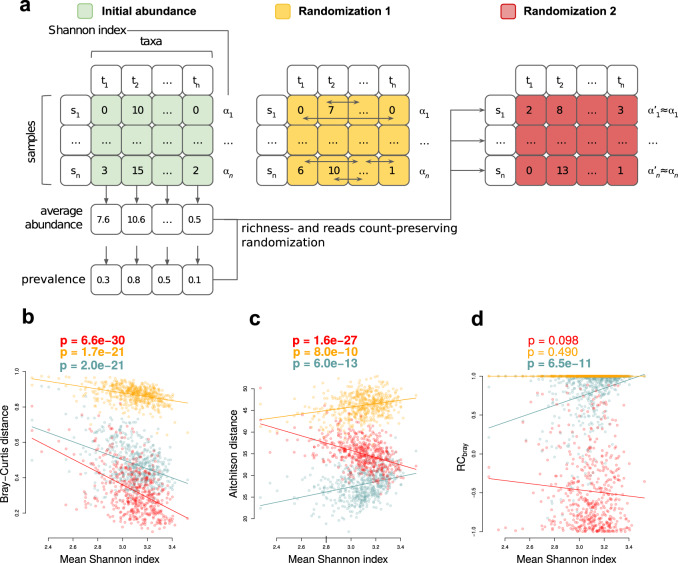
Investigation of computational components in the relationship between alpha and beta diversity. **a** Scheme of the two randomisation strategies (see Methods), **b**–**d** Relationship between average alpha diversity and beta diversity calculated for the 500 random pairs of samples from the FGFP cohort (*p* values obtained using linear regression). The colours denote initial data and data randomised by the two strategies (see legend for panel **a**). Alpha diversity was assessed via the Shannon metric, while beta diversity - via Bray–Curtis (**b**), Aitchison (**c**) and RC_bray_ (**d**) metrics.

One of the widely applied ways to evaluate microbiome response is to calculate beta diversity between paired samples collected before and after the intervention. As mentioned above, the aim of this work is to find the dependence of microbiome response on its initial composition. In this context, one of the problems of using beta diversity for assessing sustainability is its dependence on the alpha diversity of the compared communities, known from previous research in ecology^47^. The problem is that this dependence can have both a biological component (that is of high interest to investigate) and a purely computational one (that it is desirable to exclude). Computational component arises from the mutual dependence of alpha and beta diversities from the specific sample characteristics such as sparsity, and depends on the chosen diversity metrics. We explored the computational component on 500 sample pairs from the FGFP (Flemish Gut Flora Project) cohort^48^. By using two abundance randomisation techniques (see Materials and Methods, Fig. 1a), we showed that the computational dependence exists for two diversity metrics widely used in microbiome research - Aitchison^49^ and Bray–Curtis dissimilarities (Fig. 1b, c, randomised data).

One way to evaluate differences between communities taking account of the computational dependence on alpha diversity is the one based on the so-called null-models. The approach originally developed by Raup and Crick^50^ and adopted for ecology and microbiome analysis by Chase et al.^47^ and Stegen et al.^51^ allows one to estimate how much the observed beta diversity differs from that expected for a given alpha diversity, average taxa abundance and prevalence in communities. We have chosen the variant of the Raup–Crick metric based on Bray–Curtis dissimilarities proposed by Stegen et al. and denoted as RC_bray_^51^. It varies from −1 to 1, where −1 corresponds to completely identical communities, and 1 - to the most distinct ones. It can be seen that, in contrast to the beta diversity, such an estimate does not carry a computational dependence on the alpha diversity (Fig. 1d, randomised data). This is unsuprising, since RC_bray_ was developed specifically to correct for the computational dependence and the procedure for its calculation even includes a randomisation similar to the “randomisation 2” in our analysis. This fact makes RC_bray_ the metric of choice for our purposes. For validation purposes, the analyses involving adjustment for alpha diversity were additionally conducted using other beta diversity metrics.

### Dependence of response on the baseline microbiome composition

We explored the microbiome response to dietary interventions in five independent studies resulting in eight different interventions lasting from 2 to 10 weeks (see Materials and Methods). Briefly, the interventions were: high fibre diet (HFD)^44^, fermented dairy product fortified with probiotic (FDP)^45^, resistant starch from potatoes (F.POT)^37^, resistant starch from maize (F.HIM)^37^, inulin from chicory root (F.INU)^37^, accessible corn starch (F.ACC)^37^, maltodextrin (MAL)^35^ and non-meat diet (NM)^43^. The overall dataset included 1242 microbiomes from 641 individuals. The countries where the studies took place were USA, Russia and New Zealand (Supplementary Fig. 1); the metadata collected in the studies are summarized in the Supplementary Table 2.

**Fig. 2:**
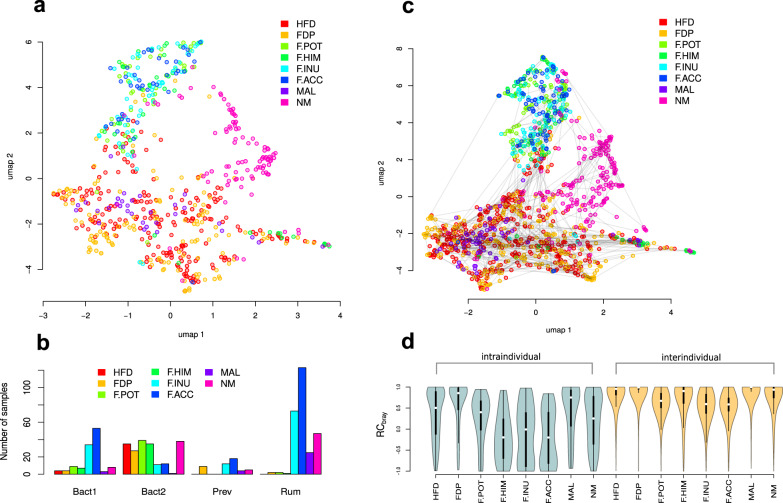
Variation of microbiome composition in the analysed data. **a** Distribution of baseline samples in the species abundance space visualised using the UMAP algorithm^65^ (*n* = 641 samples). Colours denote interventions (abbreviations are disclosed in the text). **b** Distribution of baseline samples by enterotypes. **c** Microbiome composition shifts occurring during the interventions visualised in the species abundance space using the UMAP algorithm. Grey lines connect paired samples from the same subject collected before and after the intervention (*n* = 1242 samples). **d** Intra- and interindividual variation calculated using RC_bray_ metric for each of the analysed interventions.

As expected, baseline taxa abundances strongly differed across the studies (Fig. 2a). However, enterotyping in the context of the large FGFP cohort (see Materials and Methods) showed that each original FGFP enterotype (named Bact1, Bact2, Rum and Prev as in ref. ^52^) included samples from more than one study (Fig. 2b). Therefore, all further tests including the combined set of the interventions were conducted with the correction for the intervention (in most cases - by including it as a random effect in mixed effect models); this affects the variants of tests both with and without stratification by enterotype.

The microbiome response was measured as RC_bray_ within the pairs of samples collected before and after the intervention. In all investigated studies, the microbiome response to dietary intervention varied notably and exceeded the variance of the mean response in different studies (intrastudy variance 0.3722 ± 0.0612, interstudy variance of the mean response 0.0080, Fig. 2c, d). The variance was not correlated neither with the mean response nor with the intervention time period (linear regression, *p* > 0.1). The response was not correlated with any of the baseline sample factors collected in the studies either (Supplementary Table 2).

**Fig. 3:**
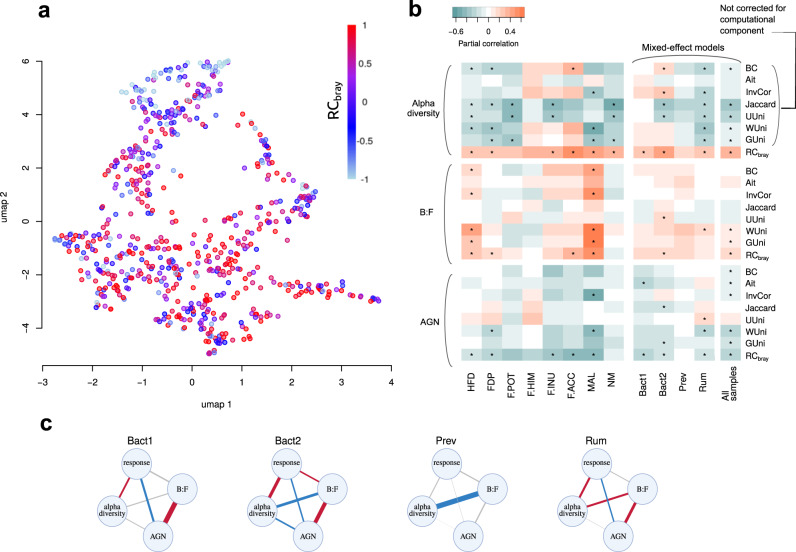
Associations between microbiome response to interventions and baseline microbiome composition. **a** Distribution of baseline samples in the species abundance space coloured in the value of response - RC_bray_ between samples before and after the intervention (UMAP algorithm). **b** Seven alternative beta diversity metrics were used for the response calculation: Bray–Curtis (BC), Aitchison (Ait), generalised UniFrac (GUni), weighted UniFrac (WUni), unweighted UniFrac (UUni), Jaccard and inverse Pearson correlation (InvCor). Colours denote partial correlation coefficients between the response and each of the analysed features. Asterisks denote significant associations (partial correlations, FDR < 0.05). Inconsistency for associations with alpha diversity between response metrics had been expected due to the computational component. **c** Enterotype-wise partial correlation networks between the response, baseline alpha diversity, AGN and B:F. The edges width is proportional to the absolute correlation coefficient. Blue colour denotes significant negative associations, red - significant positive and white – insignificant (significance estimated using partial correlations, FDR < 0.05).

The overall variance of the response did not correlate with the microbial composition at the first time point (dbRDA, *p* = 0.3017, *R*^2^ = 2%, Fig. 3a). However, the effect was observed when stratification by enterotype was performed: significant associations were revealed for the Rum (dbRDA, *p* = 0.0003, *R*^2^ = 1.2%, *N* = 212, *p*) and Bact2 (dbRDA, *p* = 0.0403, *R*^2^ = 8.0%, *N* = 195) enterotypes, while for the Prev (dbRDA, *p* = 1, *R*^2^ = 1.5%, *N* = 67) and Bact1 (dbRDA, *p* = 0.4031, *R*^2^ = 1.4%, *N* = 167) enterotypes the results remained non-significant. When the studies were analysed separately, association was detected in 5 of 8 interventions: HFD, FDP, F.POT, F.INU and MAL (dbRDA, *p* < 0.1, *R*^2^ - from 1% to 5%).

As for distinct microbiome features, dependence of response from three variables - baseline alpha diversity, *Bacteroidetes:Firmicutes* ratio (B:F) and mean number of genes per organism in the community (AGN, average gene number) - were analysed using partial correlation network due to potential correlations between the predictors (correlation between two components are computed with the correction for all the others, with intervention used as a random effect, see Methods). Baseline AGN was negatively associated with the response, while alpha diversity and B:F - positively (partial correlations, FDR < 0.05). The same effects were observed for most of the interventions analysed separately (partial correlations, FDR < 0.05, Fig. 3b). Enterotype stratification showed that associations of AGN and alpha diversity with the response were observed in each enterotype except for the Prev (partial correlations, FDR < 0.05, Fig. 3b, c); association with the B:F ratio was detected only in the Bact2 enterotype (partial correlations, FDR < 0.05, Fig. 3b, c). Since partial correlation networks include a correction for alpha diversity, we were able to validate the results for B:F and AGN using seven other beta diversity metrics for response calculation (Bray–Curtis, Aitchison, generalised UniFrac, weighted UniFrac, unweighted UniFrac, Jaccard and inverse Pearson correlation) (Fig. 3b). According to the analysis type including all samples the most reproducible was negative association with AGN (observed for all five weighted metrics), while positive association with B:F ratio was reproduced using three metrics.

Since the estimate of the AGN from 16S rRNA data might be quite imprecise, we performed additional validation of partial correlation results using “shotgun” metagenomic dataset related to a dietary intervention^53^. The intervention included 126 subjects who consumed blended cooking oils or refined olive oil for 8 weeks (three time points were collected: baseline, 2 weeks and 8 weeks). For both time intervals (2 weeks and 8 weeks), we confirmed negative association of the response with AGN (partial correlations, *p* = 0.0024 and 0.0025, respectively) and positive association with alpha diversity (partial correlations, *p* = 0.0084 and 0.0096) (Supplementary Fig. 2). For B:F ratio, no significant associations were observed. The validity of our AGN measure was supported by its high correlation with the average genome size estimate calculated using MicrobeCensus^54^ (linear regression, *p* < 2·10^−16^, Supplementary Fig. 3).

As for the associations of the response with the baseline abundance of individual microbial species, there were 52 significant taxa for combined interventions, 55 - for at least one enterotype under enterotype stratification and 37 - for at least one intervention when the stratification was conducted by intervention (Supplementary Table 3, linear mixed effect regression and simple linear regression, FDR < 0.05). Almost all significant results with enterotype stratification were identified for the Rum and Bact2 enterotypes, several associations - for Bact1 and zero - for Prev (the latter fact was possibly due to its lower number of samples). The majority of results were enterotype-specific: 28 associations were unique to Bact2 and 14 - to Rum, while 13 associations were shared among more than one enterotype. The most reproducible associations were negative dependence of response with the abundance of unclassified species from *Blautia* genus (detected in three enterotypes and four interventions) and *[Ruminococcus] gnavus* (detected in two enterotypes and six interventions). Among the enterotype-specific associations, the strongest were negative dependencies of response from unclassified *Bacteroides* abundance in Bact2 enterotype and with unclassified (*Clostridiaceae*/*Ruminococcaceae*) - in Rum enterotype (“the strongest” here means the one characterised by the maximum number of separate interventions where it was observed, Supplementary Table 3). We also repeated this analysis with the adjustment for alpha diversity, keeping in mind that we will lose taxa associated with alpha diversity (Supplementary Table 4). However, in this case, we will be able to assess reproducibility of the results using various beta diversity metrics to response calculations. The majority of the significantly associated taxa were metric-specific, with the largest number of associations being detected for RC_bray_. The only association that was replicated for all weighted metrics was a positive response to [Eubacterium]-biforme abundance in the Bact2 enterotype and overall mixed-effect model.

### Microbiome response potential

According to our observations, some microbiome features associated with the response were shared between distinct interventions. In this connection, we proposed the following assumptions and definitions:

Assumption 1: There exist microbiome markers that partly determine the response of the microbiome a priori, regardless of the intervention type.

Definition 1: *Response potential* - the component of the response that is determined solely by the individuals’ initial internal microbiome characteristics.

Thus, the observed response to an intervention is represented by the superposition of the response potential and the intervention-specific component.

Definition 2: *Landscape of response potential* - the distribution of response potential in a multidimensional space of taxa abundances.

Assumption 2: Areas of the landscape with low response potential would be characterised by increased sample density in cohorts with sufficiently large sample sizes and one sample per subject.

The latter assumption is based on the ergodicity property, which is used for macroecological communities^55^. We propose to further extend this property for microbiomes in the sense that during the adult period of life of a generally healthy individual, its microbiome can with a certain probability take on a configuration that is close enough to almost any of the compositions observed in other individuals (of the same age range). Then, such property of ergodic systems as “time average is equal to the average over space” can be interpreted as the ability to assess the stability of a single community a priori based on its position relative to the communities of other subjects in a sufficiently large cohort.

Proceeding from Assumption 2, the response potential can be estimated from its position relative to samples of other individuals in a multidimensional space of taxa abundances. One way to do this is to calculate the average beta diversity between a given sample and the samples of a relatively large number of other individuals from the same enterotype. We performed such calculations for each baseline sample in each intervention using the FGFP cohort samples and all other baseline samples as a reference (see Materials and Methods).

**Fig. 4:**
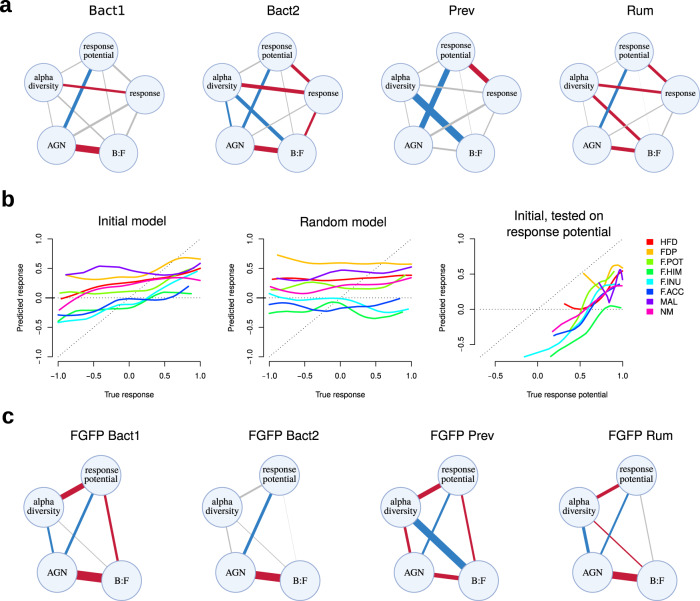
Associations of microbiome response potential with the baseline microbiome composition features and response to the interventions. **a** Partial correlation networks between the response potential, response, baseline alpha diversity, AGN and B:F calculated in each enterotype. The edge's width is proportional to absolute correlation coefficient. Blue colour denotes significant negative associations, red - significant positive and white – insignificant (significance estimated using partial correlations, FDR < 0.05). **b** Relation between predicted and true response (or response potential) for different interventions. Predicted response was calculated based on baseline species abundances using XGBoost machine learning algorithm. **c** Partial correlation networks between the response potential, baseline alpha diversity, AGN and B:F calculated in each enterotype on the FGFP cohort.

We introduced response potential to the partial correlation network (along with AGN, alpha diversity and B:F ratio) to explore its relation to response and baseline microbiome characteristics. A significant positive correlation between response and response potential was observed both in combined data and in all enterotypes except for the Bact1 (partial correlations, FDR < 0.05, Fig. 4a). These observations support our assumptions stated above. Among the associations with baseline microbiome composition, the response potential replaced the response in association with AGN, but not with the alpha diversity and B:F (Fig. 4a). The correlation of response potential with the response and AGN were validated using other beta diversity metrics (Supplementary Fig. 4).

We also tested the selected method of response potential evaluation using a machine learning approach. In particular, we estimated the extent to which the response component predictable by the initial microbiome composition is correlated with our estimate of the response potential. We trained a gradient tree boosting model (XGBoost) to predict the response based on baseline microbiome content (Materials and Methods, Fig. 4b). The model showed better cross-validation performance characteristics compared to a random model (Fig. 4b, Table 1). Then we tested the model initially trained to predict response using the values of response potential instead of response in the testing set. This substitution improved the performance characteristics for the majority of studies (Fig. 4b, Table 1), showing that the proposed approach to calculate response potential is reasonable. In other words, the result of the machine learning algorithm, which is a complex function of baseline taxa abundance, converged with the assessment of the response potential proposed by us via the position of the analysed microbiome relative to others. The robustness of our response potential concept was further supported by the fact that a response potential calculated for the second time point samples was highly correlated with the above-described calculations from the baseline values suggesting that this characteristic is only slightly affected by the analysed interventions (Supplementary Fig. 5).

**Table 1:** Average model quality characteristics obtained in 50 iterations of cross-validation trained and tested on initial data, on the data with randomly permuted sample names, and after substitution of response values with the response potential values in the testing sets.

Model	Caret R2^a^			Does the mean caret R2 increase while testing on response potential compared to the initial model?
Intervention	Initial model	Random model	Initial, tested on response potential	
HFD	0.11 ± 0.07	0.01 ± 0.02	0.15 ± 0.08	yes
FDP	0.1 ± 0.09	0.02 ± 0.06	0.05 ± 0.06	no
F.POT	0.13 ± 0.15	0.04 ± 0.1	0.18 ± 0.16	yes
F.HIM	0.2 ± 0.16	0.00 ± 0.1	0.25 ± 0.18	yes
F.INU	0.45 ± 0.19	0.02 ± 0.06	0.35 ± 0.14	no
F.ACC	0.15 ± 0.19	0.04 ± 0.1	0.2 ± 0.17	yes
MAL	0.06 ± 0.16	0.00 ± 0.12	0.08 ± 0.17	yes
NM	0.09 ± 0.09	0.00 ± 0.04	0.17 ± 0.13	yes

^a^calculated as it is proposed in the caret package [72]: the square of the Pearson correlation coefficient between the true values in the test set and the predicted values.

The response potential, according to our definition, does not depend on the type or presence of intervention, and can be calculated from a single time point. To validate the associations of the response potential with microbiome features, we tested them on the FGFP cohort dataset^48^. Association with AGN was confirmed for all the enterotypes as well as for the entire cohort (partial correlations, FDR < 0.05, Fig. 4c). Associations with alpha diversity and B:F were detected for the entire cohort as well as for several enterotypes (partial correlations, FDR < 0.05, Fig. 4c).

## Discussion

Pronounced interindividual variability of response to dietary interventions has been observed in numerous studies examining diverse intervention types - from modifying a dietary pattern to introducing a certain nutrient or probiotic microorganism to diet. The measures of response were also quite different across the studies (Supplementary Table 1). Beta diversity is often used as a measure of microbiome response. However, its computational dependence on alpha diversity is rarely taken into account. In our work, we emphasised that this is an important point, especially when searching for the baseline microbiome markers of the microbiome response. In this regard, we proposed to use the R_bray_^51^ metric as a response measure, for which the above-mentioned computational dependence is much less pronounced, according to our results. Still, this metric is not flawless. One disadvantage is its dependence on the total pool and abundance of bacterial taxa in all analysed samples. On the one hand, this leads to the changes in pairwise dissimilarity between samples when they are analysed in different contexts^47^. On the other hand, average taxa abundance in the context is implicitly taken into account during metric calculation. This may be one of the reasons why we observed more associations with the baseline microbiome features for RC_bray_ metric compared to other metrics in the analyses where adjustment for alpha diversity was performed. This observation, coupled with the notable differences between the results obtained using different response metrics (Supplementary Table 4) emphasise the need for a future detailed study on appropriate response measures.

Although the investigated interventions were quite different in both duration and the expected effects on microbiome, the intrastudy variance of mean response still notably exceeds the interstudy response variance. The dependence of response on initial microbiome state was significant for the majority of interventions. However, when the interventions were combined, this dependence remained significant only when stratification into enterotypes was applied for the two enterotypes with the highest number of samples. This fact may indicate that the intervention-independent microbiome signatures of response are enterotype-specific. This is also supported by the results observed for individual taxa. Most associations were detected for a specific enterotype, despite a lower statistical power compared to the mixed-effect models over all samples. Interestingly, the main enterotype-specific signatures were negatively associated with the response and included taxa phylogenetically related to the enterotype drivers: unclassified *Bacteroides* in Bact2 and (*Clostridiaceae*/*Ruminococcaceae*) in Rum. This may indicate that in each enterotype, the stability of a sample increases, the closer it is to the enterotype centre - suggesting the latter is an attractor in a certain sense. However, to confirm this hypothesis, a deeper study of this problem is required. Several associations revealed at the level of individual taxa reproduced previously found microbiome stability markers: positively associated with the response *Prevotella copri* and negatively - unclassified *Bacteroides* (in the previous study where “shotgun” sequencing was used it was *Bacteroides uniformis*, in our study this taxon was included in the group for unclassified *Bacteroides* species)^19^.

The relationship with the average number of genes per microorganism in the community (AGN) was observed for the entire pool of samples, as well as for all enterotypes except the Prev. This may be due to a smaller number of samples in the Prev enterotype, as well as a lower compositional variation within it. Average number of genes per organism in the community can be considered as an indicator of the prevailing ecological roles in the community. It is known that a predominance of generalist taxa possessing a higher number of genes and, accordingly, a wider metabolic potential - compared to the specialist taxa - contributes to the microbiome stability^56^. The AGN, together with alpha diversity, determines the functional redundancy of the community: AGN - at the level of an individual organism and alpha diversity - at the community level. Indeed, it is functional diversity and redundancy that are considered in theoretical ecology as stability markers^57^.

**Fig. 5:**
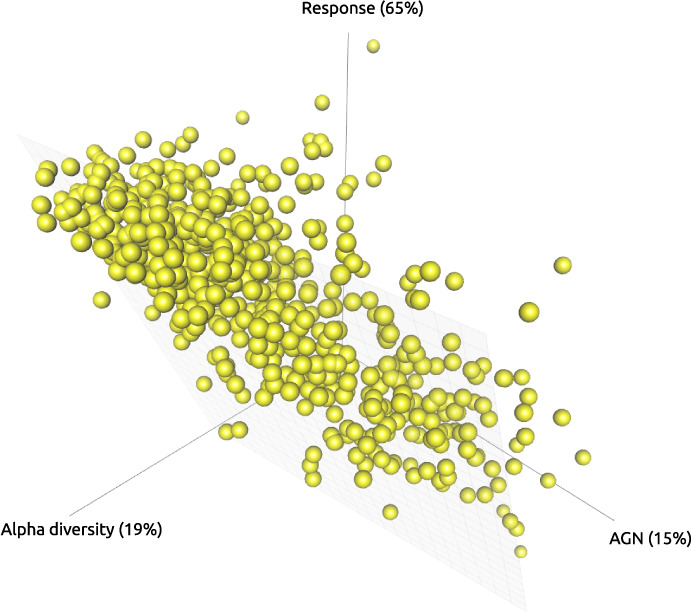
Relation between the response, baseline alpha diversity and baseline AGN. Scatterplot showing negative relationship between alpha diversity and AGN, AGN and response and positive relationship between alpha diversity and response for baseline samples from all analysed interventions (*n* = 641). Variation of data explained by each axis is given in brackets.

Interestingly, however, the association of response with alpha diversity in our study was not negative, as might be expected from the above statements and as it had been observed in several previous studies^32,34,41,43^. This discrepancy can have several causes. Firstly, we have used RC_bray_ response measure, which considers for the computational component in the alpha and beta diversity relationship, and had not previously been applied in the studies investigating microbial communities stability markers. Noteworthy, for other response metrics that did not include a correction for the computational component, the association had an opposite sign (Fig. 3b). Secondly, a negative correlation is observed between AGN and alpha diversity. This means that more diverse communities are formed by microorganisms with fewer genes (Figs. 3, 5), which also corresponds to the literature data^58^. Thus, functional redundancy is achieved through a trade-off between the number of genes per organism and the number of organisms (Fig. 5). However, we would stay cautious in drawing unambiguous conclusions about the relationship between response and alpha diversity. As we mentioned above, a further detailed study of response measures is needed to confirm and understand the nature of this relationship. As for the negative association with AGN, it was observed with multiple response metrics in different analysis types, which makes this association more reliable.

The identification of intervention-independent response markers is an important step for an assessment of an individual's microbiome stability. However, we have also looked at the problem of assessing stability from the other side. We have estimated stability based on the sample relative position to other individuals’ samples in the space of taxa abundances. We showed a high correlation of this estimate, called the response potential, with the real response in the interventional studies. In addition, using machine learning, we have isolated the part of the response that can be explained by the baseline microbiome, and have shown that for most interventions this variable correlates better with the response potential than with the response itself. However, in the partial correlation networks analysis, some of the intervention-independent associations of the response with the baseline microbiome composition persisted when the response potential was introduced into the networks. This may indicate that the method of the response potential estimation can be improved. One way of such improvement may be stratification of the acceptable microbiome configurations landscape by certain phenotypes, such as sex and age. This is particularly true when the age range is wider than in the data we used (17–65 years). For example, infants’ microbiomes will form a completely different part of the landscape. The same problem will appear when analysing subjects with a certain disease if the disease affects the microbiome composition. It is also noteworthy that the space of analysed interventional studies was generally well covered by the FGFP cohort (Supplementary Fig. 6). Therefore, we performed the calculation of the response potential in the context of the FGFP data. However, it is unlikely this will hold true for any other given study. In such cases, the reference context for calculating the response potential will have to be modified. Finally, we confirmed the associations between the microbiome response markers and the response potential on an independent cohort.

Compared to the 16S rRNA sequencing data, “shotgun” metagenomics/WGS provide a more detailed microbiome portrait, especially its functional potential, subspecies-level diversity and gene content. It might be interesting to confirm our findings on WGS data - when a sufficient number of relevant publicly available datasets of this format is accumulated. As a pilot step, we have validated them - as well as our approach for AGN calculation - using one large “shotgun” dataset (Supplementary Figs. 2, 3).

To conclude, meta-analysis of diverse dietary interventions studies suggests existence of universal baseline microbiome features defining microbiome response. One of the most common features of intervention-resistant communities is high average number of genes per microorganism in the community, likely reflecting enrichment of generalist microorganisms compared to the specialists. Reproducible specificity of response markers across enterotypes, predictability of response from the baseline location in microbiome landscape and problem of dissecting biological and computational components in alpha diversity and response relationship highlight key points to be considered during future gut microbial ecology studies.

## Methods

### Datasets description

To investigate the dependence of microbiome composition change from its initial state, we used the data from five previously published studies^35,37,43–45^. All the studies investigated microbiome response to the diet intervention using 16S rRNA gene sequencing of stool samples. For the studies where subjects underwent consequential interventions (e.g., a course of one fibre type intake followed by a course of another fibre), we picked a subset of time points corresponding to a single intervention per subject. Overall, we prepared data on eight distinct interventions where each individual was characterised by two time points in the resulting dataset - before and after the intervention. The selected interventions were:

- change of dietary pattern towards higher fibre intake for 2 weeks (HFD, N=215)^44^;
- intake of a fermented dairy product with *Bifidobacterium animalis* strain BB-12 for 2 months (FDP, *N* = 150)^45^;
- intake of fermentable fibres/placebo for 2 weeks: resistant starch from potatoes (F.POT, *N* = 43), resistant starch from maize (F.HIM, *N* = 43), inulin from chicory root (F.INU, *N* = 50) and accessible corn starch (F.ACC, *N* = 39)^37^;
- intake of maltodextrin for 3 weeks (MAL, *N* = 33)^35^;
- 2 weeks washout period followed by 4 weeks of non meat protein source intake (NM, *N* = 109)^43^.

Additionally, the FGFP dataset (*N* = 1106)^48^ including one time point per individual was processed for the purposes of:

- enterotyping;
- investigating a computational relationship between alpha and beta diversity;
- validating the dependencies discovered for the response potential.

One “shotgun” metagenomic dataset^53^ described microbiome changes during the intervention including intake of blended cooking oils or refined olive oil for 8 weeks (*N* = 126 subjects, 3 time points); it was used for:

- validation of response associations with alpha diversity, AGN and B:F;
- validation of AGN calculation.

### Data processing

For all 16S studies in the analysis, the raw data generated using Illumina MiSeq or HiSeq sequencing were downloaded from the NCBI Sequence Read Archive (ERP018192^44^, ERP109009^45^, SRP120250^35^, SRP128128^37^ and SRP166672^43^). The FGFP raw reads had been provided by the request via the European Genome Archive (EGAD00001001936). In all studies but the MAL study, the V4 16S rRNA gene region was sequenced. In MAL, V3-V4 regions were analysed, therefore, in order to unify the studies we trimmed the raw reads from this dataset using V4 primer sequences (GTGBCAGCMGCCGCGGTAA, GACTACNVGGGTMTCTAATCC) with cutadapt software^59^. Taxa abundance profiles were calculated on Knomics-Biota platform^60^ using “16S dada2 GreenGenes V4” analysis protocol, which included the following main steps:

- denoising by DADA2 algorithm^61^ implemented in QIIME2^62^;
- taxonomic classification of obtained amplicon sequence variants (ASV) with QIIME2^63^ classifier trained on preprocessed GreenGenes database (database preprocessing steps included trimming of 16S rRNA sequences using the primers given above with TaxMan^64^ software and further clustering 97% identical sequences with CD-HIT software^65^);
- microbial abundance tables at the levels of species and genus were obtained by summing the relative abundance levels of ASV included in the corresponding clade.

Only samples with >3000 reads post-denoising were included in the analysis. For HFD and FDP studies, we also excluded samples with outlying abnormal composition (e.g., those with dominating *Enterobacteriaceae*). In F.POT, F.HIM, F.INU and F.ACC interventions having 1–5 samples per time point and subject, we calculated the resulting microbiome profiles by averaging the abundances across the replicates. After preprocessing, the number of subjects per intervention was the following: HFD - 206, FDP - 130, F.POT - 43, F.HIM - 42, F.INU - 50, F.ACC - 39, MAL - 33, NM - 98, FGFP - 1,061. All samples included in the analysis are listed in Supplementary Table 5.

Filtering of relative abundance tables was done by excluding the taxa with abundance >20 reads in <20 samples. All analyses but the enterotyping (see below) were conducted at the level of species. Alpha diversity was calculated using Shannon index after rarefying all abundance tables to the minimum read count in each dataset. We calculated beta diversity using Bray–Curtis, three UniFracs, Jaccard and inverse Pearson dissimilarities after rarefaction and Aitchison dissimilarity - after substituting all zero values in the dataset by pseudocounts (0.5). The Raup–Crick metric based on Bray–Curtis dissimilarities (RC_bray_)^51^ was calculated after rarefaction (iCAMP package). As RC_bray_ depends on the overall species pool in the abundance matrix, the values of the metric for the pair of samples - for example, in the context of specific intervention and in the context of all samples from the same enterotype - can slightly differ. Therefore, we provide a description of the abundance tables used to calculate RC_bray_ metric for different analyses in Supplementary Methods. Visualisation of the relative position of samples in the taxa space was carried out by applying the UMAP algorithm with the default parameters to the matrices of the relative species abundance values^66^.

The “shotgun” dataset used for validation of partial correlation analysis results^53^ was downloaded from the NCBI Sequence Read Archive (PRJNA728374) (*N* = 378 samples). The reads were processed using KneadData (https://huttenhower.sph.harvard.edu/kneaddata/) and MetaPhlAn3 software^67^ with “-t rel_ab_w_read_stats” option to obtain the read counts. Only the taxa detected at the level of species were included in further analysis. For computational purposes, the abundance tables were rarefied to 10,000 reads per sample prior to the RC_bray_ calculation.

### Evaluation of computational component in beta and alpha diversity relation

To explore the computational component in alpha and beta diversities relationship, we used two randomisation options on 500 sample pairs from the FGFP cohort (Fig. 1a). In both cases, randomisation allowed us to obtain random taxa abundances while maintaining the alpha diversity value of the sample. Thus, the biological relationship between alpha diversity and microbiome composition was eliminated. After randomisation, we recalculated beta and alpha diversities on randomised abundance tables and used a linear model to explore the relationship between them. In this analysis, Shannon index was used as the metric for alpha diversity, while the Bray–Curtis and Aitchison^49^ dissimilarities, as well as RC_bray_^51^ - as metrics for beta diversity.

In the first randomisation variant, the names of taxa in each sample were randomly permuted, independently of other samples (Fig. 1a, “randomisation 1”). In the second variant, the procedure similar to the one used in the RC_bray_ calculation algorithm^51^ was applied (Fig. 1a “randomisation 2”). It included the following steps:

- average taxa abundances and average taxa prevalence were calculated using the whole abundance table;
- for each sample, the list of taxa names equal to the number of unique taxa in the sample were randomly sampled without replacement from the whole taxa pool with the taxa probability proportional to average taxa prevalence.
- for each sample, taxa counts equal in sum to the number of reads in this sample were randomly sampled with replacement using only taxa from the list obtained on the previous step with the taxa probability proportional to average taxa abundance.

The first randomisation gave beta diversity values between pairs of samples higher in average than the initial ones, while the second - lower ones. The first randomisation preserves the Shannon index of the sample, while after the second changes it slightly.

### Enterotyping

We performed enterotyping in the context of the FGFP cohort on the level of genera^48^. Firstly, we calculated enterotypes on genus-level abundance tables for the FGFP cohort using Dirihlet multinomial mixture models^68^ (R package DirichletMultinomial^69^). Enterotypes’ names for the FGFP cohort were matched with those described by Vandeputte et al.^52^ using the criteria listed by Valles-Colomer et al.^70^. Then, we performed classification of the samples from the interventional studies into obtained enterotypes by calculating Bray–Curtis dissimilarity between the classified sample and each enterotype medoid. The sample was assigned an enterotype according to the medoid providing the lowest dissimilarity value.

### Estimation of the mean number of genes per microorganism in the community

We estimated the average number of genes per microorganism in the community (AGN) using information from the NCBI database about prokaryotic genome assemblies (https://www.ncbi.nlm.nih.gov/genome/browse/#!/prokaryotes/). The evaluation was performed on the level of species. For all ASVs whose taxonomic assignment was resolved on the level of species (no “unclassified” term in the taxonomy), we calculated the average number of genes in the NCBI assemblies corresponding to this species (Supplementary Fig. 7). For ASVs taxonomically resolved only at the genus level (for example, “*Bacteroides* unclassified”), we performed averaging over all assemblies belonging to a specific genus. We excluded from the analysis the ASVs resolved only at the family level or higher (for example, “*Clostridiaceae* unclassified”), as well as the ASVs with taxonomies having no match in the NCBI database. Next, we calculated the weighted average number of genes per organism in the sample by multiplying the number of genes determined for each ASV by its relative abundance (read count) and dividing by the total number of reads belonging to all ASVs that were taken into account in the analysis for this sample. The obtained AGN was compared to estimation of average genome size with MicrobeCensus^54^ using “shotgun” data described above.

### Statistical analysis

Most statistical tests aimed to investigate microbiome response dependence on initial composition were performed in three variants:

- using the samples from all eight interventions in one model with the correction for the intervention;
- using samples from each enterotype separately with the correction for the intervention;
- using samples from each intervention separately.

Firstly, we analysed overall variance of response associations with baseline microbiome content using distance-based redundancy analysis dbRDA with 7000 permutations^71^ (R vegan package^72^). For dbRDA analysis, we scaled RC_bray_ metric to the [0;1] interval. Correction for the intervention was implemented through stratification of the data during permutations.

Then we analysed associations of the response with alpha diversity, B:F ratio and AGN using a partial correlation network. This analysis method was chosen because all four components have significant correlations between each other, and we are interested in the strongest ones. During a partial correlation network analysis, an association between two components *i* and *j* is calculated as a Pearson coefficient between two linear models residuals. Each linear model includes one of the analysed components (*i* or *j*) as a predicted variable and all components of the network except *i* and *j* - as predictors. Correction for the intervention was conducted by using a mixed effect linear model with intervention as a random effect instead of a simple linear model. Correction for multiple comparisons was performed using the Benjamini–Hochberg method. The partial correlation network from five components (including response potential) was constructed in the same way. For this analysis, we performed a validation using a “shotgun” dataset described above^53^.

We analysed association of individual taxa abundance with the response using a linear model after clr transformation of the abundance tables^49^. The analysis was performed at the level of species. Zero abundance values were replaced with pseudocounts (0.5). Correction on intervention was conducted by using a mixed effect linear model with intervention as a random effect instead of a simple linear model. Correction for multiple comparisons was performed using the Benjamini–Hochberg method.

Correlation of response with baseline metadata collected in studies was evaluated using a mixed effect model with intervention as a random effect or simple linear model (the former was used when the factor was available for several interventions, while the latter - when the factor was collected in only one intervention.)

### Microbiome response potential

To investigate the universality of discovered associations between microbiome composition and response, we introduced the definition of the response potential as a response component determined solely by the initial microbiome state (see Results). We proposed an approach to estimate response potential as the mean RC_bray_ value between the sample and the samples from the same enterotype belonging to a relatively large number of other individuals (we used more than 300 individuals per enterotype) (the justification can be found in the Results section). This approach allows one to estimate response potential from the data including 1 time point per individual.

### Machine learning approach to test the response potential estimate

A machine learning approach was used to determine the extent to which the response component predictable from the baseline microbiome composition is correlated with the proposed estimate of the response potential. For this purpose, we constructed a model aimed to predict the response based on baseline taxa abundances. It was constructed separately for each intervention using the gradient boosted trees (XGBoost)^73^. The arcsine square root transformed baseline relative abundances on the species level were used as predictors. The following parameters were used in the model: tree depth - 15, number of trees - 1000, learning rate (step size) - 0.3. Model quality was evaluated via a cross-validation procedure by randomly dividing the data into training and testing sets 50 times in 2:1 ratio. We performed predictors filtration and selection on each iteration based on the information in the training set. During filtration, taxa with zero abundance in >30% of the samples were removed. Predictors selection included training of 10 additional XGBoost models on the training set of the iteration, followed by evaluation of the predictors’ importance. These additional models had the same parameters and structure as the main one, except for the number of trees (300). We estimated predictors importance as its relative contribution to the improvement of the prediction in each tree in the model (xgboost R package^74^). The 30 predictors with the highest importance were chosen to construct the main model on specific iteration. To assess the quality of the resulting regression, we calculated *R*^2^ values by the method proposed in the caret package^75^ (representing the square of the Pearson coefficient between the true and predicted response) at each iteration. The average *R*^2^ values across all iterations were used as a model quality measure.

We compared the regression quality with the quality of a random model. The latter was obtained by repeating cross-validation for the abundance matrices with randomly shuffled sample names. After that, we analysed how the quality of the regression has changed when the response values were replaced by the values of response potential in testing set during cross-validation. If we observed similarity of *R*^2^ values between this and initial models in comparison to the random model, that would indicate that the proposed method of response potential calculation is a good estimate for the component of response determined by baseline microbiome state.

## Supplementary information

Supplementary Information

Supplementary Tables

## Data Availability

In this study, we re-analysed raw data from previously published datasets. Accession numbers are listed in the Materials and Methods section.

## Code availability

All preprocessed data (ASV tables) (except FGFP dataset) as well as the code to reproduce all of the analyses are available at https://bitbucket.org/natasha_klmnk/art_variation_public.
