# Supplementary material for: The hallmarks of dietary intervention-resilient gut microbiome
Source: NPJ Biofilms Microbiomes. 2022 Oct 8;8:77. doi: 10.1038/s41522-022-00342-8 (PMC9547895; doi:10.1038/s41522-022-00342-8)
Supplement: Supplementary file 1 — Supplementary Information [file 41522_2022_342_MOESM1_ESM.pdf]

Supplementary figures

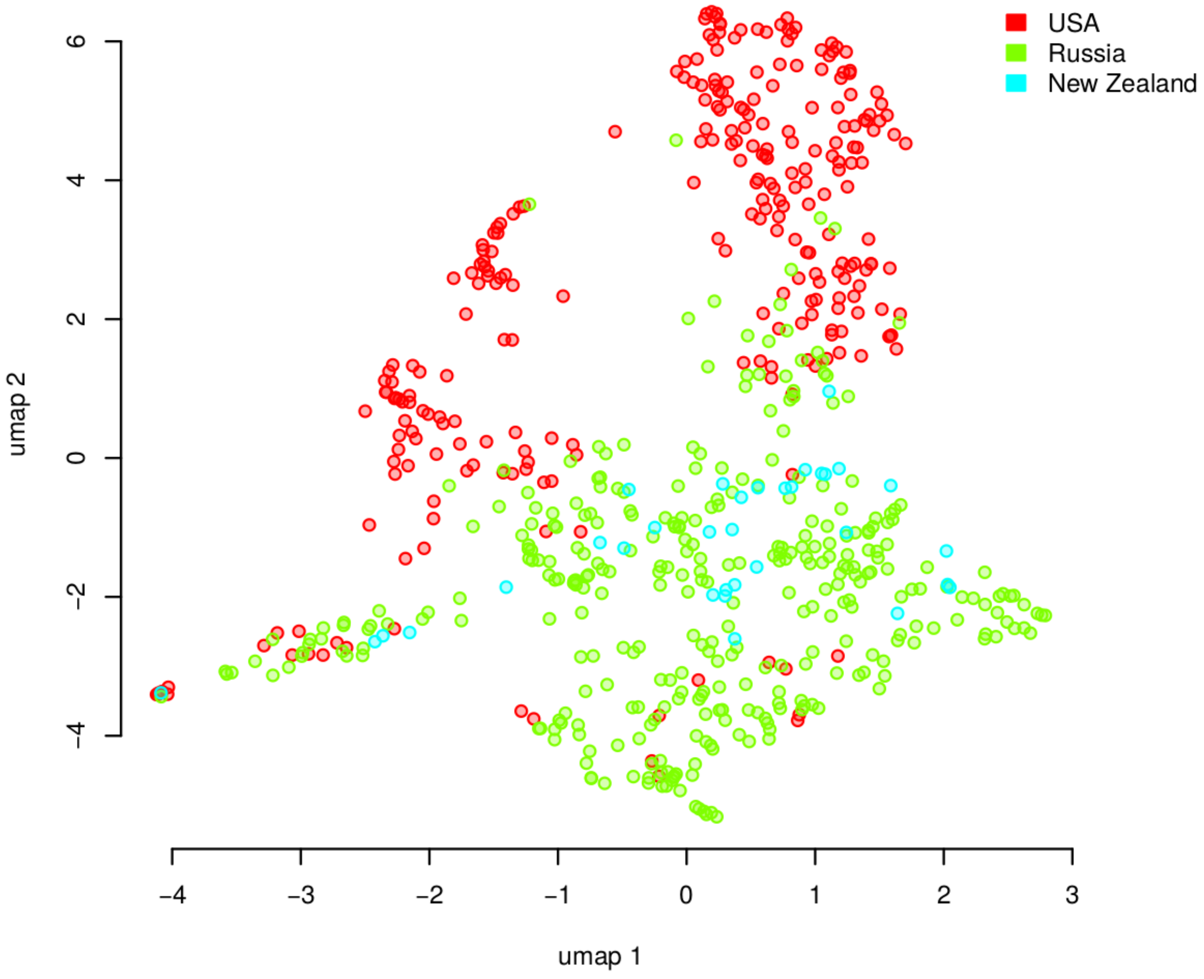

Supplementary figure 1 - Distribution of baseline samples in the species abundance space visualised using UMAP colored by country (n=641 samples).

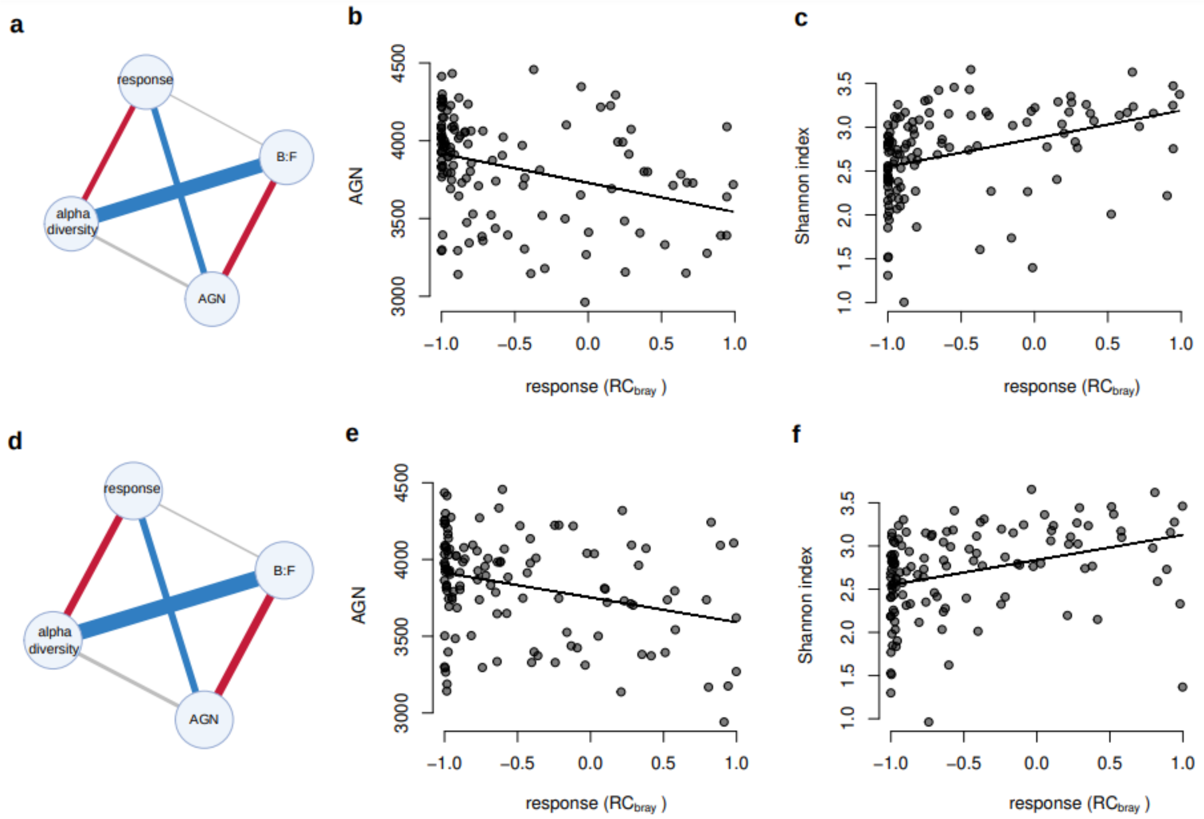

Supplementary figure 2 - Validation of partial correlation network obtained in 16S rRNA meta-analysis on "shotgun" data (N=126). a, d - Partial correlation networks between the response, baseline alpha diversity, AGN and B:F for 2 (a) and 8 (d) weeks of intervention. The edge width is proportional to the absolute correlation coefficient. Blue colour denotes significant negative associations, red - significant positive and white - insignificant (significance estimated using partial correlations, FDR<0.05). b, e - Relation between baseline AGN and response for 2 (b) and 8 (e) weeks of intervention. c, f - Relation between baseline alpha diversity and response for 2 (c) and 8 (f) weeks of intervention.

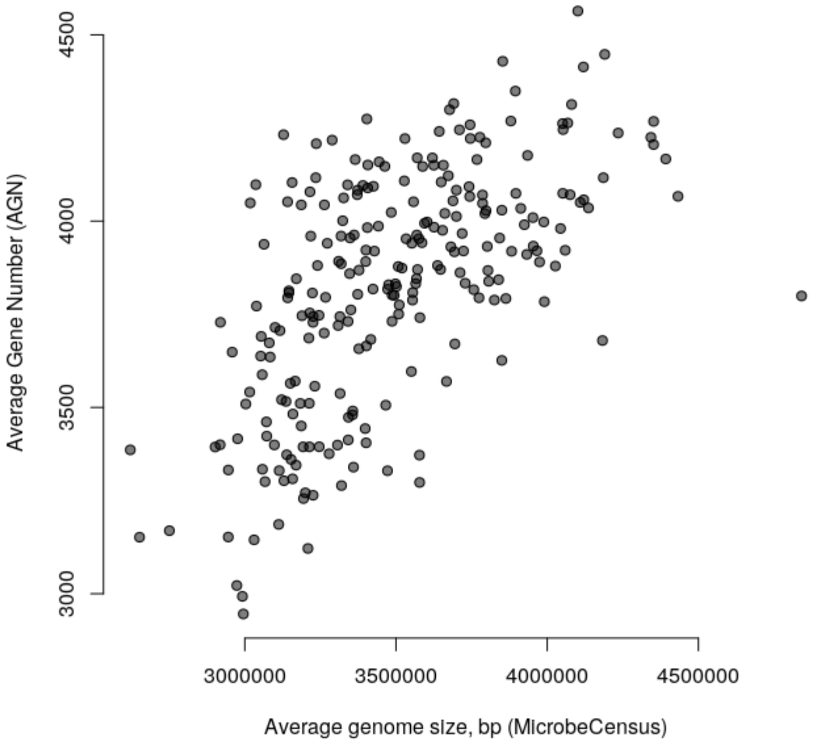

Supplementary figure 3 - Validation of AGN calculation using “shotgun” data (N=378 samples from 126 subjects). Our method of AGN calculation was compared to average genome size estimation obtained using MicrobeCensus tool intended for “shotgun” data.

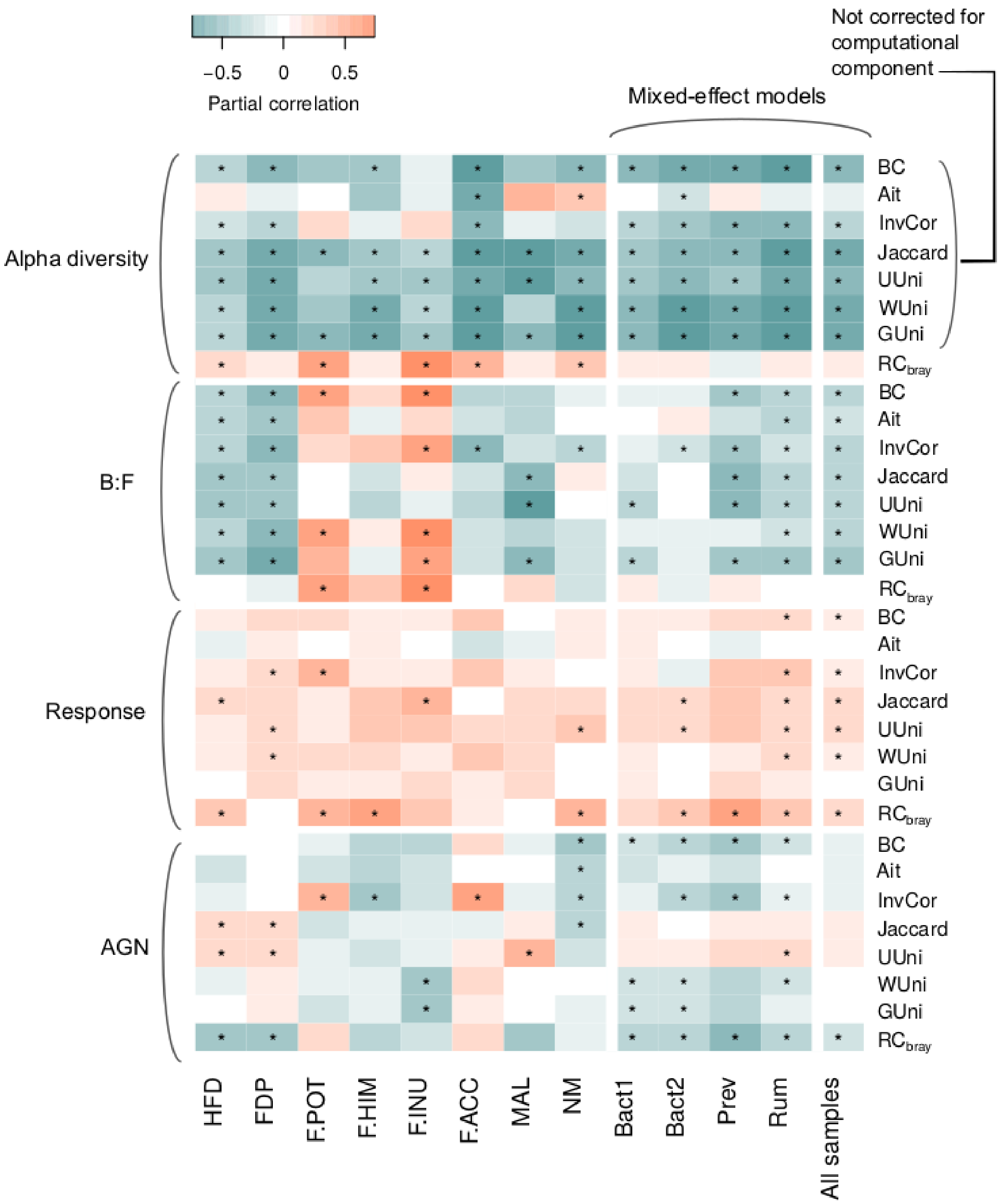

Supplementary figure 4 - Associations between microbiome response potential and each of the baseline alpha diversity, AGN, B:F and response to interventions calculated as partial correlations. Eight additional beta diversity metrics were used for the response calculation: Bray-Curtis (BC), Aitchison (Ait), generalised UniFrac (GUni), weighted UniFrac (WUni), unweighted UniFrac (UUni), inverse Pearson correlation (InvCor) and Jaccard. Colours denote partial correlation coefficients between the response potential and each of the analysed features. Asterisks denote significant associations (partial correlations, FDR<0.05). Inconsistency for associations with alpha diversity between metrics was expected due to the computational component.

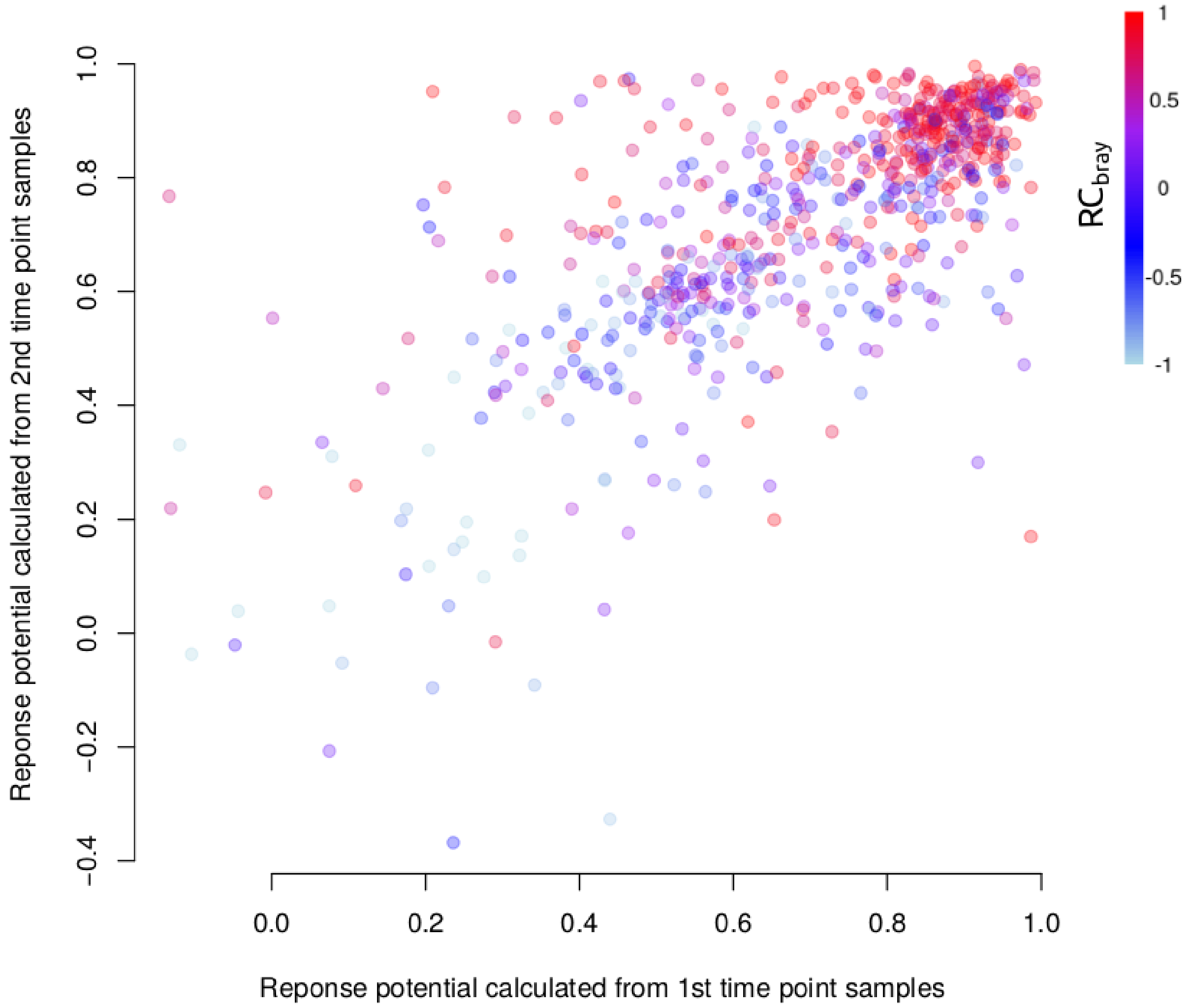

Supplementary figure 5 - Relation between response potential calculated for samples at the 1st time point and at the 2nd time point. Samples are colored by the value of observed response - RC_{bray} between the samples before and after the intervention (N=641 pairs).

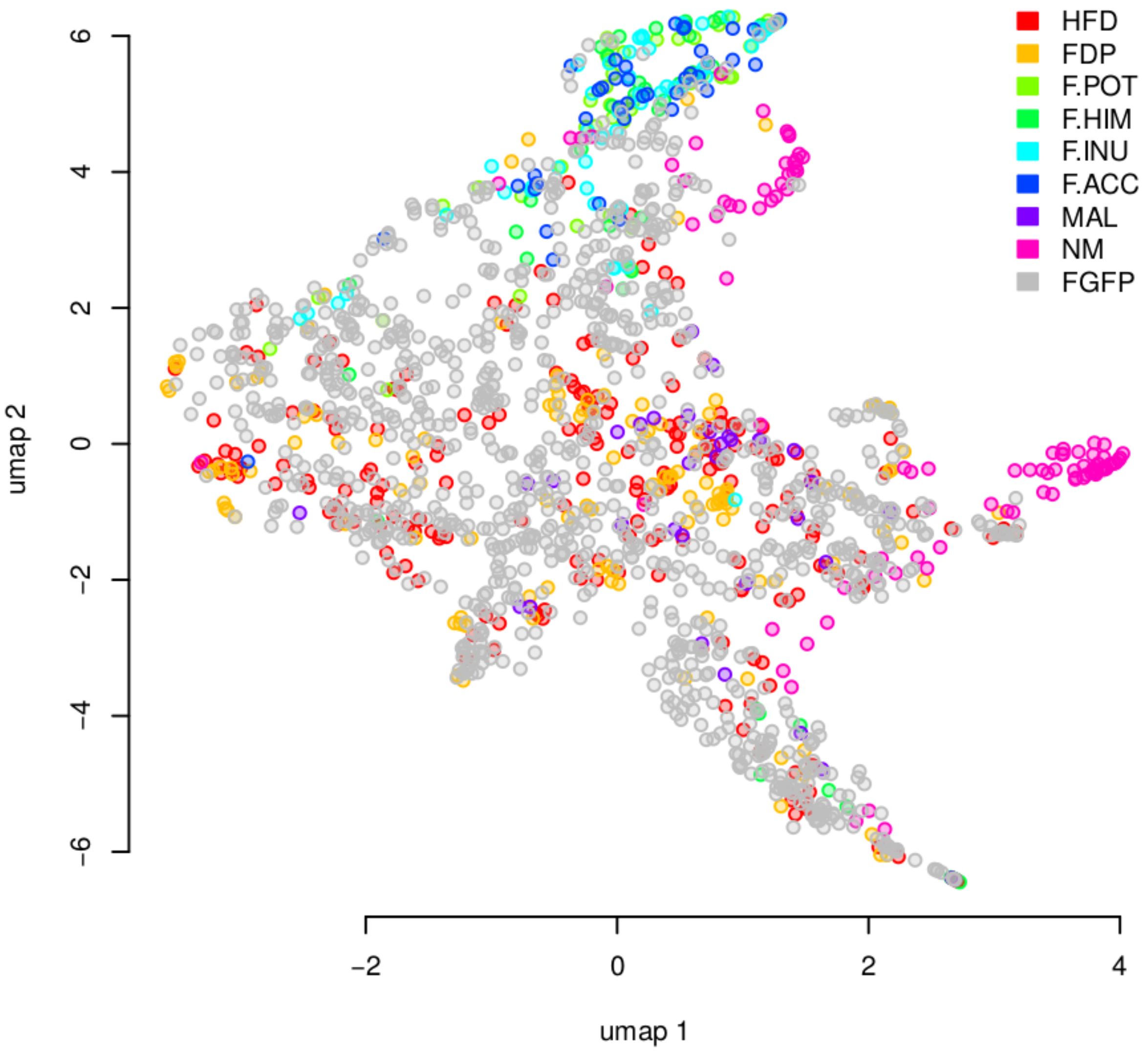

Supplementary figure 6 - Distribution of baseline samples from interventional studies and FGFP samples in the species abundance space visualised using UMAP [52].

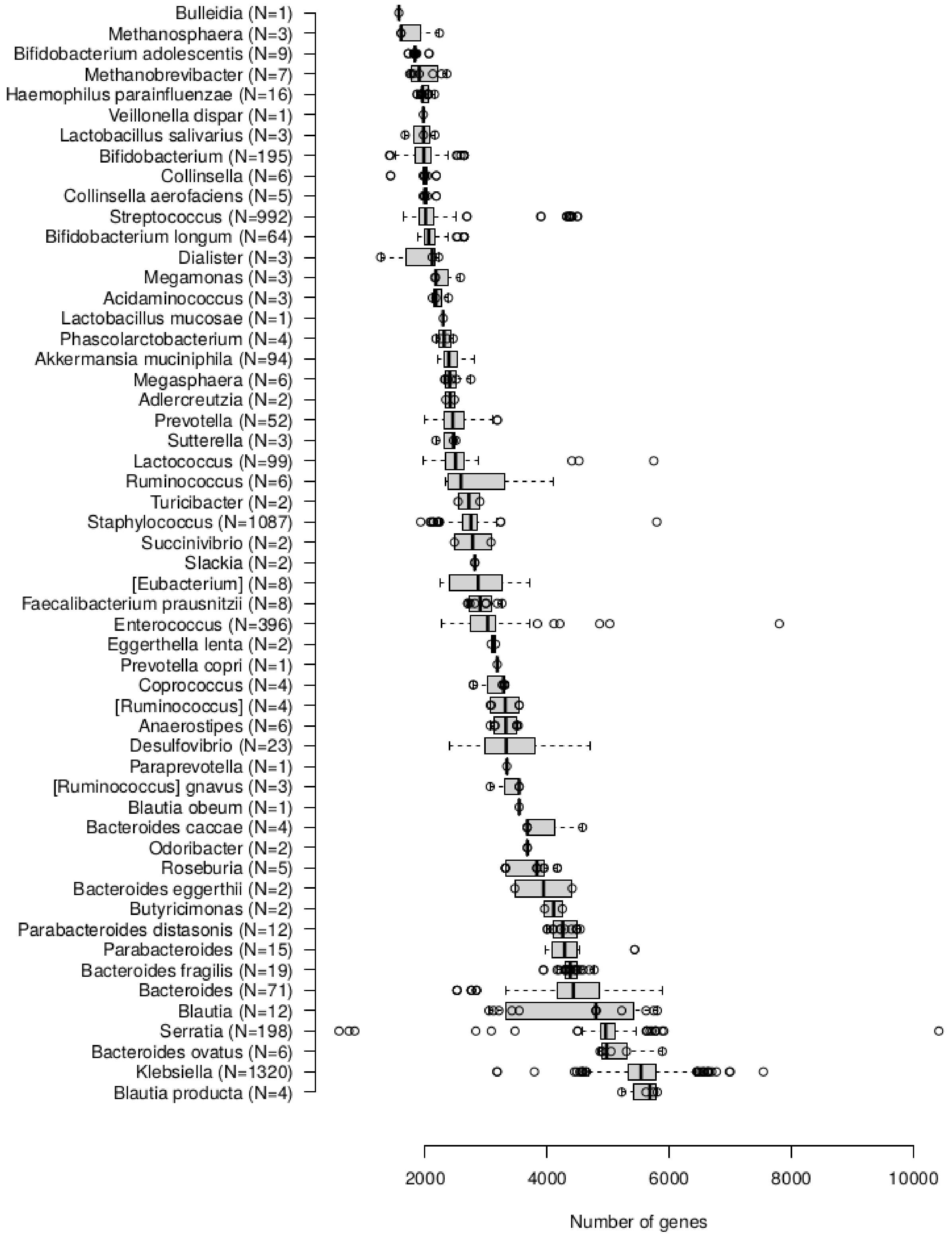

Supplementary figure 7 - Gene number for different taxa used to calculate AGN from the NCBI database. Number of genomes used to calculate mean gene count for each taxon is given in brackets. For taxa with N<20 individual data points are shown above the boxplot.

Supplementary methods

Abundance tables used to calculate RC_{bray} metric in different analysis sections:

- for the calculation of RC_{bray} between paired samples before and after the intervention, we used one abundance table per intervention;
- for the calculation of RC_{bray} between all samples before the intervention (dbRDA for all studies, see below), we used an abundance matrix including all baseline samples from all interventions;
- for the calculation of RC_{bray} between all samples from the specific enterotype before the intervention (dbRDA for all studies with enterotypes stratification, see below), we used an abundance matrix including all baseline samples belonging to the analysed enterotype from all interventions;
- for the calculation of RC_{bray} between all samples from the specific enterotype before the intervention in the context of the FGFP cohort (response potential calculation, see below), we used an abundance matrix including all baseline samples belonging to the analysed enterotype from all interventions and FGFP cohort;
- for the calculation of RC_{bray} between all samples from the specific enterotype in the FGFP cohort (response potential calculation for validation on FGFP cohort, see below), we used an abundance matrix including all samples belonging to the analysed enterotype from FGFP cohort.

Supplementary tables legends

Supplementary table 1. Studies that investigated the dependence of microbiome response to the dietary interventions on baseline microbiome composition. The "-" sign denotes that the factor associations with the response have not been analysed. Abbreviations used in the table: FMT - faecal mass transplantation, OTU - operational taxonomic unit, LDL - low density lipoproteins, HDL - high density lipoproteins.

Supplementary table 2. Additional per-sample baseline parameters available online (as NCBI SRA metadata or Supplementary material) for studies used in meta-analysis: summary and relation to microbiome response (RC_{bray}).

Supplementary table 3. Significant associations between microbiome response to the interventions and species baseline abundance according to three analyses types: mixed-effect models on all data, mixed effect models with enterotypes stratification and linear models applied for separate interventions.

Supplementary table 4. Significant associations between microbiome response to the interventions and species baseline abundance according to three analyses types: mixed-effect models on all data, mixed effect models with enterotypes stratification and linear models applied for separate interventions with the correction on the alpha diversity for eight different response metrics: RCbray, Bray-Curtis (BC), Aitchison (Ait), generalized UniFrac (GUni), weighted UniFrac (WUni), unweighted UniFrac (Uuni), inverse Pearson correlation (InvCor) and Jaccard.

Supplementary table 5. Samples included in the analysis.
